# Atherosclerotic Plaque Component Segmentation in Combined Carotid MRI and CTA Data Incorporating Class Label Uncertainty

**DOI:** 10.1371/journal.pone.0094840

**Published:** 2014-04-24

**Authors:** Arna van Engelen, Wiro J. Niessen, Stefan Klein, Harald C. Groen, Hence J. M. Verhagen, Jolanda J. Wentzel, Aad van der Lugt, Marleen de Bruijne

**Affiliations:** 1 Biomedical Imaging Group Rotterdam, Departments of Medical Informatics & Radiology, Erasmus MC, Rotterdam, the Netherlands; 2 Department of Imaging Science and Technology, Faculty of Applied Sciences, Delft University of Technology, Delft, the Netherlands; 3 Department of Biomedical Engineering, Erasmus MC, Rotterdam, the Netherlands; 4 Department of Radiology, Erasmus MC, Rotterdam, the Netherlands; 5 Department of Nuclear Medicine, Erasmus MC, Rotterdam, the Netherlands; 6 Department of Vascular Surgery, Erasmus MC, Rotterdam, the Netherlands; 7 Department of Computer Science, University of Copenhagen, Copenhagen, Denmark; INSERM U894, Centre de Psychiatrie et Neurosciences, Hopital Sainte-Anne and Université Paris 5, France

## Abstract

Atherosclerotic plaque composition can indicate plaque vulnerability. We segment atherosclerotic plaque components from the carotid artery on a combination of *in vivo* MRI and CT-angiography (CTA) data using supervised voxelwise classification. In contrast to previous studies the ground truth for training is directly obtained from 3D registration with histology for fibrous and lipid-rich necrotic tissue, and with 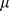
CT for calcification. This registration does, however, not provide accurate voxelwise correspondence. We therefore evaluate three approaches that incorporate uncertainty in the ground truth used for training: I) soft labels are created by Gaussian blurring of the original binary histology segmentations to reduce weights at the boundaries between components, and are weighted by the estimated registration accuracy of the histology and *in vivo* imaging data (measured by overlap), II) samples are weighted by the local contour distance of the lumen and outer wall between histology and *in vivo* data, and III) 10% of each class is rejected by Gaussian outlier rejection. Classification was evaluated on the relative volumes (% of tissue type in the vessel wall) for calcified, fibrous and lipid-rich necrotic tissue, using linear discriminant (LDC) and support vector machine (SVM) classification. In addition, the combination of MRI and CTA data was compared to using only one imaging modality. Best results were obtained by LDC and outlier rejection: the volume error per vessel was 0.9
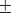
1.0% for calcification, 12.7
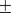
7.6% for fibrous and 12.1
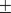
8.1% for necrotic tissue, with Spearman rank correlation coefficients of 0.91 (calcification), 0.80 (fibrous) and 0.81 (necrotic). While segmentation using only MRI features yielded low accuracy for calcification, and segmentation using only CTA features yielded low accuracy for necrotic tissue, the combination of features from MRI and CTA gave good results for all studied components.

## Introduction

Atherosclerotic disease of the carotid artery is common in the elderly population, and is a major cause of cerebral ischemia [1], [2]. The underlying mechanism is the rupture of atherosclerotic plaque with subsequent embolisation of thrombus and/or plaque material in the cerebral circulation. Clinical manifestations and fatal outcomes are most often associated with plaques of American Heart Association (AHA) type IV, V and VI [3]. On MRI these are characterized by presence of a lipid or necrotic core (LRNC) and possibly calcifications (type IV–V) or a possible surface defect, hemorrhage or thrombus (type VI) [4]. These characteristics as found in carotid histology have been related to recent symptoms [5], [6], and measurements of tissue components from MRI have been related with future events [7], [8]. Prevention of (recurrent) cerebral ischemia is the goal of pharmacological or surgical treatment. Currently the decision for surgical treatment such as carotid endarterectomy or carotid artery stenting is based on the degree of stenosis, but incorporating non-invasive measures of plaque composition is expected to improve the selection of patients that will benefit from surgical intervention [9]–[11].

Non-invasive identification of different plaque components is possible both with magnetic resonance imaging (MRI) [12]–[14] and CT-angiography (CTA) [15], [16]. Manual component segmentation and quantification in MRI is time-consuming and subject to inter- and intraobserver variability [13], [17]. Automated segmentation methods that are accurate and robust are therefore essential to perform large scale studies that can determine the clinical relevance of plaque composition, and to be able to incorporate these measures into daily clinical practice if this is deemed advantageous. In this paper we perform automatic segmentation of plaque components using a combination of MRI and CTA images and evaluate the advantage of combining those imaging modalities.

Automated methods that segment plaque components have been developed previously, but these show some limitations in the accuracy for different components, and use possibly inaccurate or biased training and evaluation methods. Considering the accuracy for different plaque components, in MRI good results have generally been obtained for quantification of fibrous tissue and LRNC, but except for the results reported in [18] a low accuracy for calcification has been found [19], [20]. CTA on the other hand, provides a good estimation of calcium volume, while the differentiation between LRNC and fibrous tissue is more challenging due to a large overlap in Hounsfield values [15], [16]. A combined analysis of MRI and CTA may be beneficial for accurate quantification of all plaque components [21], [22].

The use of possibly inaccurate or biased training and evaluation methods results from the difficulty to obtain an accurate ground truth. Supervised pattern classification is commonly used as part of segmentation methods, and voxelwise classification has also been applied to successfully segment plaque components from MRI [18]–[20]. These techniques require a known voxelwise ground truth for classifier training. The ground truth may be obtained from manual segmentations [20], but these may be inaccurate due to overlapping intensities between classes and inter-observer and intra-observer variability [18], [19]. Histology sections are considered to be more objective [23], but it is difficult to accurately align these with *in vivo* scans due to tissue deformations that occur during surgical plaque excision and histology processing. Histology-guided manual annotations have been used as well [18], [19], but may introduce a bias toward the *in vivo* scan data [24]. In this paper we choose to use the more objective information directly obtained from histology, while trying to account for misregistration during classifier development.

Image registration between histology and *in vivo* data is a topic of interest in many applications [25]–[27], and using the registration with histology as a ground truth for *in vivo* pattern classification is a challenging problem [28], [29]. For atherosclerotic plaque, image registration between histology and *in vivo* data has mostly been done by manually selecting corresponding slices followed by rigid 2D registration [15], [16], [18], [19]. Non-rigid 3D registration has also been used, to allow rotation of the *in vivo* image orientation with respect to the histology slicing direction, and to compensate for in-plane deformations in histology [30]. Although this does allow for the correct rotation angle, it remains difficult to obtain voxelwise correspondence for the vessel wall and plaque components due to the large deformations that occur owing to plaque excision and histology processing.

Another approach to handle registration inaccuracies when registered data is used to train a classifier, is to account for inaccurate sample labels during the training phase. Several ways to cope with inaccurate labels have been proposed. One approach is to detect outlier samples and reject those samples from the training set. An overview of methods for outlier rejection is given by Hodge and Austin [31]. Another approach is to adjust the weight or label of samples with an uncertain label. Bouveyron and Girard [32] used prior clustering to detect samples with inconsistent labels and took these inconsistencies into account during supervised modeling. Prior clustering has also been used to create fuzzy labels that indicate a membership probability for each class [33]. In this way outlier samples get a low membership value for the class they belong to according to the hard label. Thiel [34] showed that classifiers based on such ‘soft’ labels are robust against label noise by artificially adding different levels of noise to soft labels. The approaches above use distances in feature space to determine soft labels. In our case we have additional knowledge on the probability that labels are accurate. At the border between plaque components errors are more likely to occur than in the center because of misregistration between histology and *in vivo* images. In addition, locations where the histology and *in vivo* images align well provide more accurate labels than locations that are less well registered. We evaluate two approaches that use this information to modify the sample labels and/or weights and compare to using the original hard labels and a standard way of (Gaussian) outlier rejection. In addition, registration between different MRI sequences, and between CTA and MRI, is important for accurate classification. We will present an approach for these registrations.

In this paper we perform plaque component segmentation in *in vivo* imaging data. We combine MRI and CTA scans to differentiate between calcification, fibrous tissue and lipid-rich necrotic tissue. The main contributions of this paper are 1) the evaluation of different approaches for training on histology data which account for registration errors, 2) the combination of MRI and CTA imaging features for plaque characterization and the evaluation of their performance, and 3) optimization of a 3D registration framework to match *in vivo* MRI and CTA with histology. Together these steps present a framework for quantification of plaque components in *in vivo* data, by training on registered histology. A preliminary version of this paper has been presented previously at a conference [35]. The current paper presents more ways of handling registration accuracy during training, has a more elaborate evaluation and discussion, and includes a comparison between MRI and CTA.

## Materials and Methods

This section is structured as follows. After the *Ethics statement* we first we describe the data, which consist of 13 arteries (13 patients) that are all imaged with corresponding histology, *ex vivo* MRI, 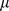
CT and *in vivo* MRI and CTA. Image registration of the *in vivo* images and histology sections, in order to obtain a ground truth to train the segmentation method, is subsequently described. Next we describe the design of the classifiers, including the different ways of handling registration inaccuracies for training. Finally, the experiments for evaluation are presented. Final registered data of all subjects (CTA, MRI, histology, labeled ground truth and *in vivo* wall segmentation) will be made available upon request.

### Ethics statement

This study was approved by the Medical Ethical Committee of the Erasmus Medical Center. Written informed consent was obtained from all subjects.

### Data

Fifteen patients (all male, age 68
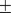
9 years) who were scheduled for carotid endarterectomy (CEA) were selected for this study and gave informed consent. Nine had an ischemic stroke, five had a transient ischemic accident and one was asymptomatic. A subset of this data has previously been used for the development of a 3D registration framework [30] and to develop a segmentation method on *ex vivo* MRI [36]. Due to incomplete imaging data and a low quality of histology two patients were excluded, leaving thirteen datasets for the analysis. Before CEA, patients underwent *in vivo* MRI (Signa Excite (3 Tesla), GE Healthcare, Milwaukee, USA) and CTA (Sensation 16 (n = 4)/Sensation 64 (n = 9), Siemens, Erlangen, Germany) scanning. MRI was made one day prior to CEA, and CTA 38
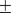
26 days earlier. We used four MRI scans that were made before contrast administration (2D-T1w, 2D-PDw, 2D-TOF and 3D-T1w), and one 3D-T1w scan 4.6
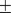
3.4 minutes after intravenous administration of gadofosveset (Vasovist, 0.03 mmol/kg body weight, Bayer Schering Pharma AG). Details are provided in Table 1. Due to the better performance of contrast-enhanced T1w scans to differentiate plaque tissues compared to T2w scans, no T2w scans were used [37]–[39]. CTA images were made with a standardized contrast-enhanced protocol [40] and had a resolution of 0.27
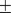
0.05 mm in-plane with a slice thickness of 0.9
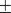
0.1 mm and a slice distance of 0.5
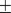
0.1 mm. After registration of the MRI and CTA scans (Section *Image registration*) manual annotations of the vessel wall were made on the registered *in vivo* scans. Annotations were based on a combination of CTA, PDw MRI and postcontrast T1w MRI, with visual inspection of the other in vivo MR sequences.

**Table 1:** MRI settings.

	Repetition time (ms)	Echo time (ms)	Flip angle	In-plane resolution (mm)	Slice thickness	Slice distance
2D-T1w Fast Spin Echo	425±77	12.1±1.1	90°	0.41±0.07	1.5	1.5
2D-PDw Fast Spin Echo	4635±284	17.2±1.9	90°	0.41±0.06	1.5	1.5
Fast Time of flight	15.3±1.2	3.4±0.3	40–60°	0.91±0.11	2–3	1.5–2
3D-T1w Gradient Echo (Pre- and postcontrast)	15.3±0.3	3.15	16°	0.61±0.05	0.8–1	0.4–0.5

As previously published [30], to facilitate registration of the *in vivo* data to histology, *ex vivo* MRI (3D-T1w Gradient Echo, 0.1×0.1×0.1 mm, Signa Excite, GE Healthcare) and 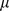
CT scans (18×18×18 µm, Skyscan 1072, Skyscan, Belgium) of the excised plaque were made. In addition photographs of the specimen were taken every 1-mm interval during histology slicing, called ‘block-face’ images (15
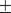
1×15
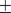
1 µm). Histology sections were taken every 1-mm interval (1.8×1.8 µm) and stained with Elastica von Gieson staining (Merck, Germany). To obtain ground truth segmentations the vessel wall was manually segmented in histology, and divided into fibrous and lipid-rich necrotic regions. The ground truth for calcification was obtained by thresholding the 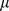
CT at a fixed value for all scans [36]. Based on histology quality, 11
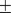
4 histology slices with registered *in vivo* images were included per subject (range 3–17 slices).

### Image registration

Our registration framework is an extension of the 3D registration of CTA with histology as described by Groen et al. [30]. That method registered CTA to histology image data using the following steps. First, a 3D histology stack is created by non-rigid registration of histology slices to a stack of ‘block-face’ photographs taken during sectioning. CTA is registered to 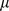
CT using isotropic scaling based on annotated landmark points in both imaging modalities, which are mainly calcium spots. To align 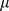
CT with *ex vivo* MRI, and *ex vivo* MRI with the 3D histology stack, a rigid transformation based on manually annotated landmarks is applied. Subsequently the 3D histology stack is deformed in-plane to match the *ex vivo* vessel wall, annotated in the *ex vivo* MRI, using a B-spline model [41] that maximizes mutual information (MI) [42], [43] of both image intensity and vessel wall annotations.

**Figure 1:**
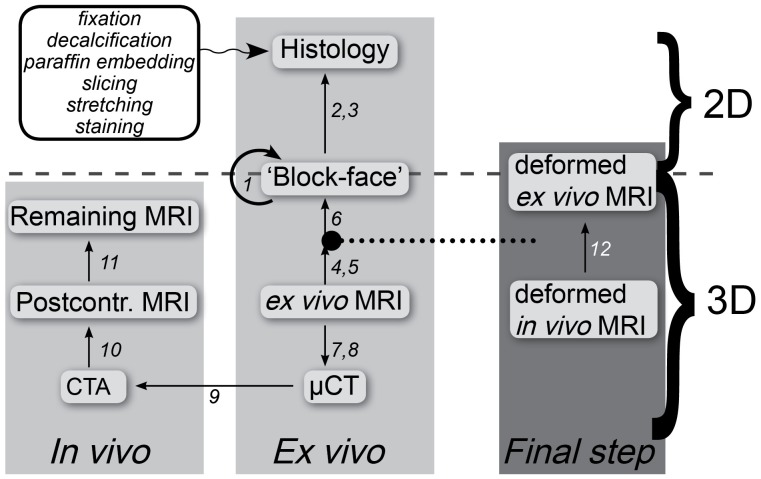
Overview of the framework for histology processing and registration. The light gray blocks show registration of *in vivo* MRI and CTA with histology, via *ex vivo* MRI and 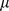
CT. The large dot with a dotted line indicates the space to which all images are transformed. After these transformations the registration step in dark gray block is done to directly optimize the registration of *ex vivo* with *in vivo* data. The arrows point from the fixed to the moving image. Numbers in this figure refer to registration steps with a detailed description in Table 2.

We made a number of modifications to this framework. Firstly, we added *in vivo* MRI. All MRI scans were rigidly registered to the postcontrast 3D-T1w scan, and this scan was rigidly registered to the CTA. These registration steps were based on mutual information of image intensity and made use of a mask around the vessel that was annotated in the fixed images (CTA and postcontrast MRI). Secondly, compared with Groen et al. the registration of histology and *in vivo* data was refined in two ways. 1) In the registration of CTA to 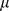
CT isotropic scaling was replaced by a thin-plate spline deformation [44], to account for deformations that occur during plaque excision. This was based on landmarks only (7.8
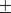
1.6 landmarks per plaque), by fitting an approximating thin-plate spline with relaxation factor of 0.1 [45]. 2) After registration using all previously mentioned steps (light gray area in Figure 1), the *ex vivo* MRI was deformed to match the *in vivo* postcontrast MRI. This was done based on maximization of the sum of MI of image intensity, MI of manual segmentations of the lumen and MI of the outer vessel wall with a B-spline model. Similar to the registration of *ex vivo* MRI with histology, a multiresolution scheme with 4 resolution levels and a final B-spline control point spacing of 2 mm was used. Optimization was done using adaptive stochastic gradient descent optimization [46]. The resulting transformation was applied to the ground truth segmentations (histology and 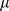
CT), to obtain a better overlap of the vessel wall in the ground truth and the *in vivo* data. The toolbox elastix [47] was used for all registrations, in combination with MeVisLab for rigid point-based registration and Python for scripting. A summary of all steps of our modified registration framework is given in Figure 1 and Table 2, and a more detailed description and evaluation can be found in [30] and [36]. The effect of the two refinements mentioned above is evaluated in *Registration results*.

**Table 2:** Settings for the different registration steps.*

	Fixed image	Moving image	Def.model	Information	Comp. time	Manual time
1	Stacking of block-face images, by registering each slice to its adjacent slice		Rigid	Landmarks	Few sec per slice	∼2–3 min per slice
2	Block-face	Histology	Rigid	Landmarks	Few sec per slice	∼2–3 min per slice
3	Block-face	Histology	B-spline	MI of intensity, lumen mask and outer wall mask	∼2–3 min per slice	∼15 min per slice (including composition in histology)
4*	*Ex vivo* MRI	Block-face	Rigid	Landmarks	∼10 sec	∼5 min per 3D volume
5*	*Ex vivo* MRI	3D histology	Rigid	MI of intensity, lumen mask and outer wall mask	∼1 min	∼15 min per 3D volume (histology annotation from step 3)
6	*Ex vivo* MRI	3D histology	in-plane B-spline	MI of intensity, lumen mask and outer wall mask	∼3–4 min	- (Uses annotations from steps 3 and 5)
7	*Ex vivo* MRI	*μ*CT	Rigid	Landmarks	0.5–1 min	∼5 min per 3D volume
8	*Ex vivo* MRI	*μ*CT	Rigid	MI of intensity	0.5–1 min	-
9	*μ*CT	CTA	Thin-plate spline	Landmarks	∼10 sec	∼5 min per 3D volume
10	CTA	Postcontrast T1w MRI	Rigid	MI of intensity within mask	∼0.5 min	∼2–3 min per 3D volume
11	Postcontrast T1w MRI	Other MRI images	Rigid	MI of intensity within mask	∼0.5 min	∼2–3 min per 3D volume
12	Deformed *in vivo* postcontrast T1w MRI	Deformed *ex vivo* MRI	B-spline	MI of intensity, lumen mask and outer wall mask	∼5–6 min	∼10 min per 3D volume

* For registration the inverse transformation of steps 4 and 5 was applied to the *ex vivo* MRI. Def. model = deformation model, Comp. time = computation time, MI = Mutual Information.

### Classifier design

Regions of interest of all *in vivo* MR images were corrected for intensity inhomogeneities using N3 [48], and normalized by setting the mean intensity to 0 and the standard deviation to 100 within these selected regions. A set of 24 image features was calculated for each voxel: the intensities in the normalized 3D-T1w (pre- and postcontrast), T1w, PDw and TOF images, these images blurred with a Gaussian filter (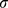 = 1 mm), the gradient magnitude and Laplacian at the same scale, the original CTA intensity, the Euclidean distances to the lumen and outer vessel wall, and the product of these distances. Intensity, first and second order derivatives and distances have previously proven to be effective [18], [20], [36]. The product of the two distances was added to enable a linear separation between LRNC and fibrous tissue, which better prevents the lipid-rich necrotic core from touching the lumen or outer vessel wall border. Together these distance features represent both wall thickness and the voxel location relative to the lumen and outer wall. For training the distances were based on the deformed histology segmentation, for testing on the distance to the manual *in vivo* contours. All images (ground truth and features) were resampled to 0.25×0.25 mm in-plane using cubic B-spline interpolation, such that they had a resolution in the order of the *in vivo* CTA.

**Figure 2:**
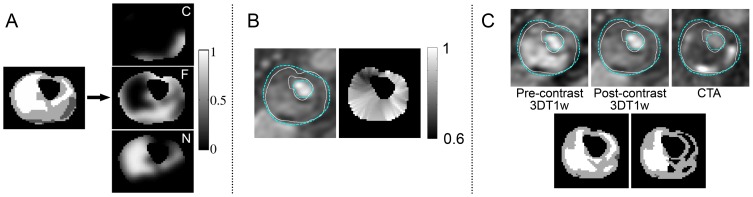
Different ways of handling registration accuracy in training. A. Soft labels for each class are derived by blurring the original segmentations. In this example 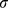 = 0.5 mm and the soft labels of the three classes sum to the Dice overlap between histology and *in vivo* data in each voxel (0.93 in this slice). In the hard segmentation dark gray is calcification (C), light gray fibrous tissue (F) and white lipid-rich necrotic tissue (N). B. Sample weights are determined by the distance between lumen and outer wall contours in histology (white line) and the *in vivo* data (blue dashed line). C. Outlier rejection: Based on 10% outlier rejection on the combination of all 13 vessels, the black areas in the right bottom figure would be rejected as outliers. In this slice mainly lipid/necrotic voxels from the right half of the section are considered outliers in feature space.

To account for registration inaccuracies, which lead to inaccurate training labels, we compared three approaches:

1. Uncertainties in the ground truth were taken into account by two mechanisms. First, the binary ground truth segmentations (calcification (C), lipid-rich necrotic tissue (LRNC) and fibrous tissue (F)) were blurred with a Gaussian filter with standard deviation 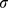 (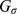), followed by normalizing the sum of the three components to 1. This creates soft labels that indicate a probability of belonging to each of the three components, where points close to component boundaries get a similar, lower, probability for multiple components. Second, since the reliability of the labels depends on the registration accuracy, we estimated registration accuracy. Hereto the Dice overlap between the vessel wall segmentation in histology and registered MRI/CTA was calculated for each slice [49]. Slices were subsequently weighted by their registration accuracy by multiplying the normalized soft labels by Dice*^n^*. Here 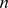 is an exponent, where with larger 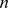 the difference in contribution of slices with low or high Dice overlap becomes larger. The final labels assign a weight to the samples, such that samples close to region boundaries or from slices with a low registration accuracy contribute less to the classifier than samples with a more certain ground truth. In our experiments, we determined the optimum value for 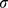 and *n* using cross-validation on the training set (Section *Evaluation*). In the equation below 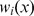 is the sample weight for class 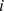 at voxel 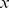, with 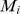 the binary mask for class 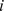.
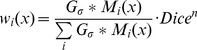
(1)
2. Approach 1 uses the same degree of blurring at all locations to obtain soft labels, and assigns a higher or lower weight to an entire slice based on registration accuracy. In case one part of the section is aligned more accurately than the other, a more local weighting of registration accuracy would be appropriate. To achieve this we calculated for each voxel the Euclidean distance to the lumen and the outer vessel wall, in both histology and the *in vivo* scans. The sample weight 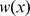 (eq. 2) was then defined as a function of weight based on outer wall (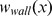) and lumen registration accuracy (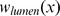), where the weight was determined to be 1 for a difference between the histology and *in-vivo* contour of 0 and 0 for a difference of 5 mm, linearly scaled between these values. The ratio of the two weights was determined by the relative distance of the voxel to the lumen and outer wall (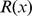):
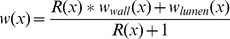
(2)

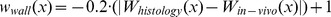


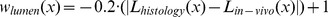


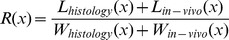
where 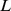 indicates the distance to the lumen and 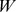 the distance to the outer wall. An example is shown in Figure 2B.
3. The third approach is Gaussian outlier detection, which excludes samples that are outliers in feature space without taking into account the position of these samples in the original image or the registration accuracy. For each of the three components, 10% of the samples was rejected. A Gaussian target distribution was modelled to the data. The mean and standard deviation for each class were robustly estimated by iteratively reweighing the samples by their distance to the (previously estimated) mean [50]. Outliers can be in misregistered areas for which the image characteristics do not correspond to the class label, but also variations in image intensity for certain scans, or imaging artifacts can be rejected. We choose to reject 10% of the samples in outlier rejection expecting this would be a good balance between not discarding too many samples and at the same time being sure that all outliers are rejected. For an example see Figure 2C.

For classification, a linear discriminant classifier (LDC) and support vector machine (SVM) classifier were used. The LDC has been used successfully in previous studies [20], [36]. The definition is as follows [51]

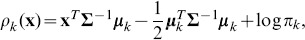
(3)where 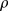 is the posterior probability, 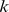 are the classes, 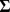 the pooled covariance matrix, 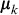 the class means, 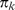 the class prior probabilities, and 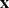 the feature vector to classify. Each sample was assigned to the class with the highest posterior probability. As LDC is a relatively simple, non-flexible classifier, we used a support vector machine (SVM) with a radial-basis function (RBF) kernel for comparison. Compared to LDC, SVM is more flexible and it has proven successful in many applications. The classification problem is solved as [52]:
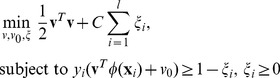
(4)

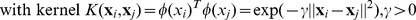
with 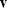 the SVM classifier and 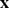 the feature vector. 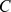 is the penalty parameter of the error term that trades-off between minimizing misclassification and maximizing the margin, 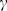 the kernel radius, 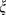 the misclassification weight and 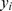 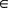 {−1,1} the sample label. The decision boundary is then defined as:
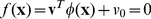
(5)New samples are labelled by thresholding 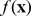 at 0. Multiclass classification was done by combining different one vs one classifiers. For classifier development and evaluation the Matlab toolbox *prtools* [53], and *libsvm* [52] were used.

### Evaluation

Leave-one-out experiments were performed in which repeatedly 12 subjects were used for training and the 13*^th^* for testing. Classifiers were trained on 10% of all voxels that were within the vessel wall in both the histology segmentation and the *in vivo* wall segmentation, and tested on all voxels within the *in vivo* vessel wall. The same 10% of samples were extracted for training for each experiment.

For labeling approach 1 with ground truth blurring and weighting with Dice*^n^*, 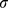 values equal to 0, 0.25, 0.5, 0.75, 1, 1.5 and 2 mm were evaluated, and *n* was varied from 0 to 39 with intervals of 3, with a separate cross-validation within the training set of 12 subjects, again by leave-one-subject-out experiments. For each combination of 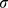 and *n* the absolute error of the calculated volumes (% of the vessel wall) of all three components with respect to the ground truth volumes was averaged over all slices of the 12 subjects, and the 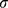 and *n* that corresponded to the lowest error were chosen. Using the same approach the penalty parameter *C* and kernel radius 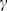 were optimized in the training set for all SVM classifications. Here, the features were normalized to have zero mean and a standard deviation of 1.

Classification with LDC and SVM was performed using the four different types of sample labels: 1) original hard labels, 2) labels obtained by ground truth blurring and weighting by the Dice overlap, 3) local weights obtained using the contour distances and 4) Gaussian outlier rejection. All voxels within the *in vivo* segmented vessel wall were classified. The results were evaluated by comparing plaque component volumes as a percentage of the vessel wall with histology, both per subject and per slice. For each classifier, the results per slice were tested for statistical significant differences between the four different approaches. The absolute errors of the three components were averaged as they are strongly related, and compared using Friedman analysis, with post-hoc Tukey-Kramer testing to account for multiple comparisons.

To evaluate performance when classification is based on a single imaging modality, voxel classification was repeated using only MRI and only CTA features, both with and without the distance features. For completeness, also classification using only distance features was evaluated using the same approach. Finally, an experiment was carried out to indicate which features are most relevant for classification. Forward feature selection with LDC accuracy as the evaluation criterion was performed for all three combinations of two components. All voxels of all 13 subjects were used, with their corresponding hard label.

## Results

### Registration results

**Figure 3:**
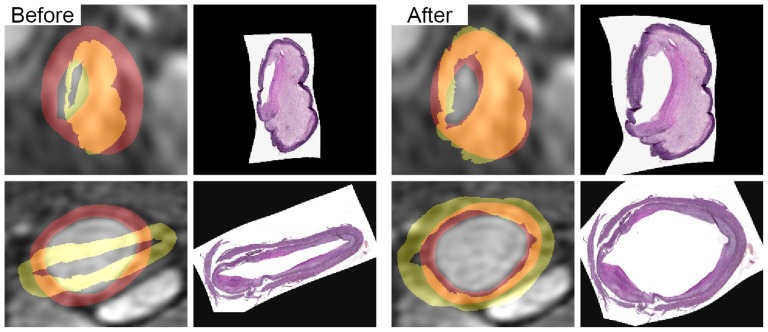
Two registered image slices. Examples are presented before and after applying the final deformation step as depicted in the right column of Figure 1. In yellow the deformed histology vessel wall is shown, in red the *in vivo* vessel wall overlaid on the postcontrast MRI scan. In the orange regions they overlap. The Dice overlap for the top image increases from 0.59 to 0.86, for the bottom image from 0.28 to 0.44.

Registering CTA to 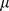
CT with a thin-plate spline deformation instead of the isotropic scaling as was used in [30], showed that in cases with large deformations an improved match was obtained (visual inspection). When deforming the *ex vivo* MRI vessel wall to match the *in vivo* MRI vessel wall (dark gray right column in Figure 1), the Dice overlap increased from 0.61
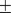
0.18 (range 0.14–0.88) to 0.77
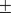
0.12 (range 0.31–0.95). Applying this deformation to the histology segmentations increased the Dice overlap between the histology vessel wall and *in vivo* vessel wall from 0.57
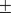
0.18 (range 0.11–0.87) to 0.67
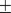
0.16 (range 0.22–0.94). The final mean wall distance between histology and *in vivo* data was 0.87
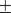
0.63 mm for the lumen and 0.67
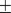
0.39 mm for the outer wall. As this error is in the order of several voxels we can assume that voxelwise correspondence was not obtained. Two examples are shown in Figure 3.

### Segmentation results

The average bias, absolute error and Spearman rank correlations of relative component volumes with respect to the relative volumes in the ground truth (histology and 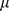
CT), per subject, are given in Table 3. The results show that for LDC good calcification classification was obtained, with errors smaller than 2% and correlation values higher than 0.75. The amount of fibrous tissue was overestimated and the amount of LRNC underestimated, but correlations >0.75 could be obtained as well. For LDC, both blurring and Dice weighting, and outlier rejection decreased the bias, but only outlier rejection reduced the absolute error, for all three components. This error was significantly smaller than for the two methods that use sample weighting, and may be slightly better than using hard labels (p = 0.06). Additionally, the improvement in absolute error using outlier rejection was significantly related with the amount of lipid per slice (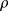 = −0.23, p<0.01). For slices with higher lipid amounts, the advantage of using outlier rejection was larger than for slices with no or little lipid.

**Table 3:** Segmentation results per subject for different approaches using MRI, CTA and distance features.*

Method	Bias (% in result - % in GT)			Absolute error			Spearman (*ρ*)		
	C	F	LRNC	C	F	LRNC	C	F	LRNC
LDC - Hard labels	−0.1±1.5	11.9±10.9	−11.8±10.8	1.0±1.1	14.1±7.5	14.0±7.4	0.91	0.87	0.84
LDC - Method 1	−0.6±1.9	8.1±14.6	−7.4±13.9	1.5±1.2	14.6±7.4	13.7±7.1	0.77	0.80	0.78
LDC - Method 2	−0.6±1.7	12.4±11.9	−11.8±11.6	1.1±1.3	15.2±7.4	14.6±7.4	0.85	0.82	0.85
LDC - Method 3	−0.2±1.4	7.6±13.0	−7.5±12.8	0.9±1.0	12.7±7.6	12.1±8.1	0.91	0.80	0.81
SVM - hard labels	0.5±2.9	−0.5±19.7	0.1±19.3	2.0±2.1	15.7±11.0	15.6±10.4	0.88	0.53	0.58
SVM - Method 1	1.2±3.9	−4.3±18.1	3.1±17.2	2.6±3.1	12.3±13.6	11.8±12.5	0.68	0.63	0.74
SVM - Method 2	−1.8±2.0	3.2±11.1	−1.4±10.6	2.1±1.7	9.1±6.7	8.2±6.5	0.63	0.73	0.79
SVM - Method 3	−1.4±3.8	−1.2±16.9	2.6±14.8	3.0±2.5	12.6±10.6	11.2±9.5	0.43	0.73	0.71

The results are compared to relative component volumes in histology.

* C = calcification, F = fibrous tissue, LRNC = lipid-rich necrotic core, Method 1 = blurring and Dice weighting, Method 2 = weighting by contour distance, Method 3 = Gaussian outlier rejection.

While classification with LDC yielded better correlations than with SVM, SVM yielded a lower bias, and, when weighting by contour distance, a lower absolute error. For SVM the three methods to handle registration errors seem to have a larger effect than for LDC. The correlations for fibrous tissue and LRNC also improved, but the results for calcification deteriorated. The differences in error between the methods with SVM, were not significant.

**Figure 4:**
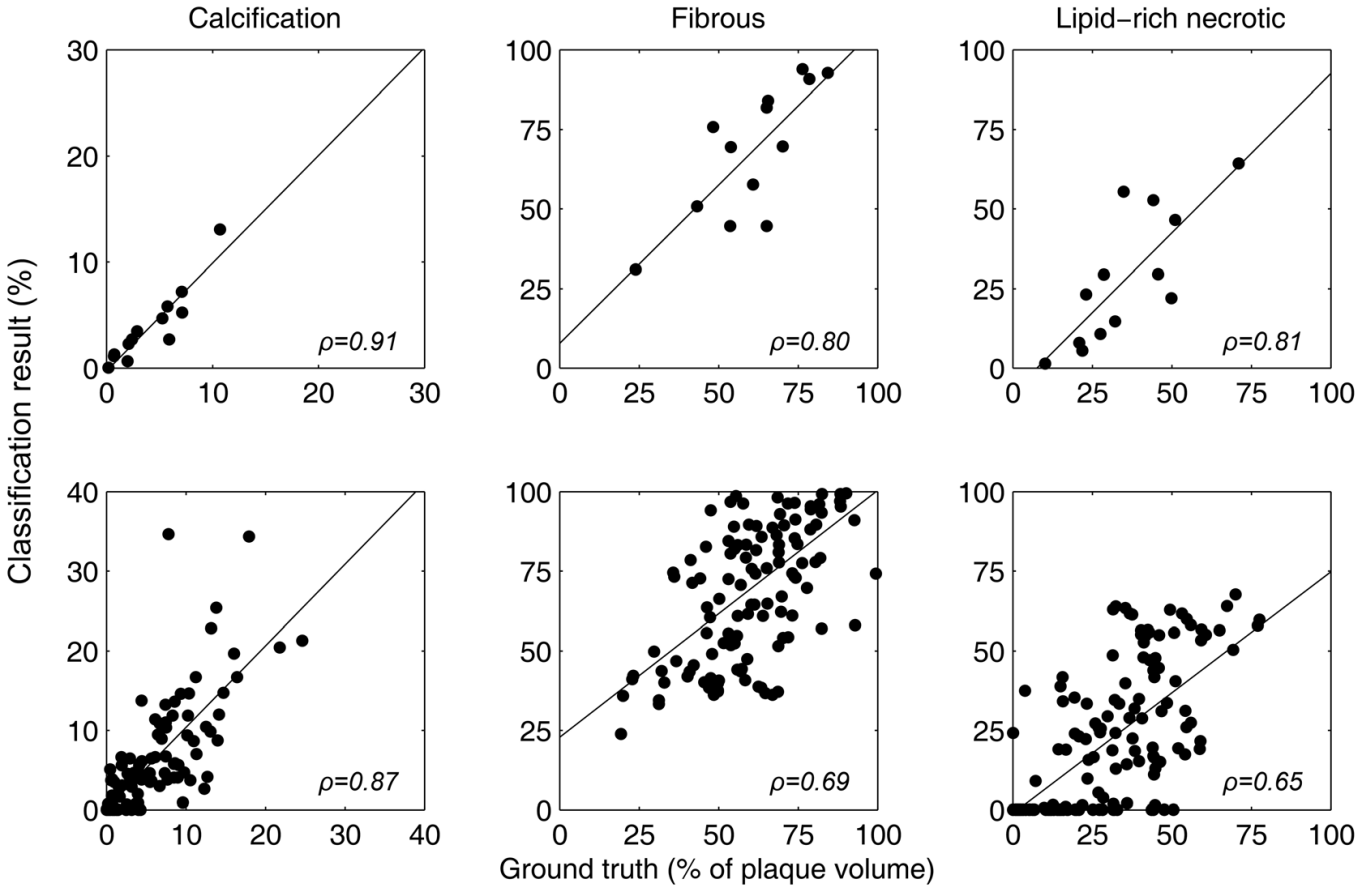
Correlation of plaque components in the ground truth and the classification result in 13 subjects. Here LDC and Gaussian outlier rejection were used. Top row: relative volumes per subject. Bottom row: relative volumes per slice.

**Figure 5:**
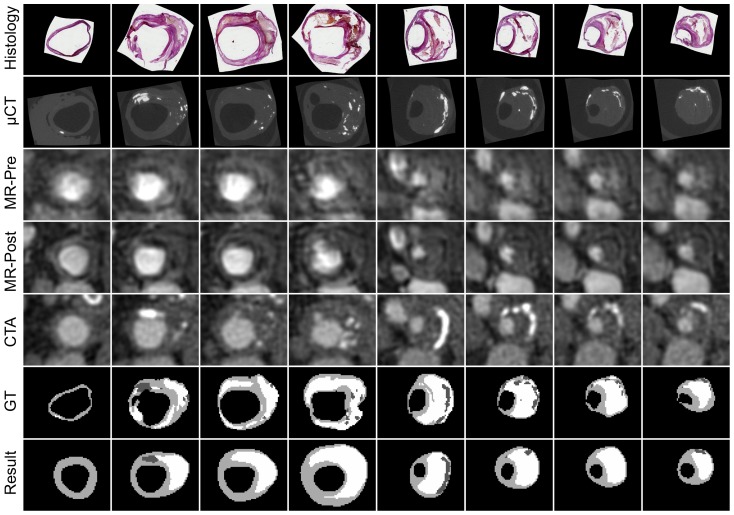
Segmentation results for one patient, using LDC and Gaussian outlier rejection. White = LRNC, light gray = fibrous tissue and dark gray = calcification. Segmentation results of all 13 patients can be found in the online movie S1.

Overall, classification with LDC and Gaussian outlier rejection lead both to a relatively low absolute error and a good correlation with histology for all three components. Therefore, additional visualizations of these results are provided. Scatter plots in Figure 4 show the correlation between the relative volume of each tissue component in histology and in the segmentation result. The segmentation results for all slices of one subject are shown in Figure 5. Segmentations of the other subjects can be found in Movie S1. The segmentations visually show acceptable spatial fidelity.

The voxelwise accuracy in the overlapping areas of the vessel wall in histology and in *in vivo* data, was 68
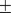
6% for LDC and hard labels and 69
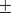
6% for LDC after outlier rejection. However, this evaluation is hampered by registration errors and the results should therefore be interpreted with caution.

For the approach with blurring and weighting with LDC the optimal values found for 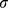 (ground truth blurring) were 0.15
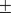
0.16 mm (range 0–0.5 mm) with *n* = 23.8
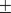
11.5 (range 12–39, ground truth weighting). Thus, no or little blurring of the ground truth segmentations was performed, but high weighting of slices based on registration accuracy was applied. This led to a skewed sample weight distribution with an interquartile range of 0.0001–0.0038–0.0411 (total range 0–0.44). For SVM 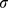 was 0.38
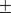
0.35 mm (range 0–1 mm), with *n* = 9.0
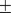
6.1 (range 0–15), and a sample weight interquartile range of 0.014–0.128–0.493 (range 0–1). Using local contour distance the obtained sample weights were 0.91
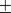
0.06 (range 0.44–1).

### MRI vs. CTA

The experiments to compare performance on MRI and CTA were also performed using LDC and Gaussian outlier rejection. The results using only MRI or CTA are given in Table 4. When only MRI features were used, calcification was underestimated and in most cases not detected. Using only the original CTA image, a good correlation for calcification was found, although the volumes were overestimated. Differentiation between fibrous tissue and LRNC was not possible. Adding distance features, however, showed a great improvement. Using the distance features only yielded plausible volume estimates for fibrous tissue and LRNC, however, adding MRI features improved the results even more.

**Table 4:** Segmentation results when only MRI, CTA or distance features are used.*

Features (n)	Bias (% in result - % in GT)			Absolute error			Spearman (*ρ*)		
	C	F	LRNC	C	F	LRNC	C	F	LRNC
MRI (20)	−3.7±3.1	13.4±14.2	−9.7±12.6	3.7±3.1	15.8±11.2	12.8±9.2	−0.17	0.60	0.67
CTA (1)	7.9±5.6	27.5±18.5	−35.4±16.3	7.9±5.6	28.1±17.4	35.4±16.3	0.90	−0.03	0
MRI + distances (23)	−3.6±3.3	9.5±13.7	−5.9±13.4	3.9±2.9	14.2±8.1	12.0±7.7	−0.05	0.81	0.79
CTA + distances (4)	−0.4±1.1	6.8±16.3	−6.4±16.1	0.8±0.9	14.7±9.1	14.2±9.2	0.94	0.77	0.71
Distances (3)	−4.0±3.3	9.0±16.7	−5.0±16.7	4.1±3.1	15.5±10.3	14.4±9.1	−0.46	0.74	0.74
*MRI + CTA (as in * *table 3* *)*	−0.2±1.4	7.6±13.0	−7.5±12.8	0.9±1.0	12.7±7.6	12.1±8.1	0.91	0.80	0.81

Outlier rejection was performed before classifier training.

* C = calcification, F = fibrous tissue, LRNC = lipid-rich necrotic core.

**Figure 6:**
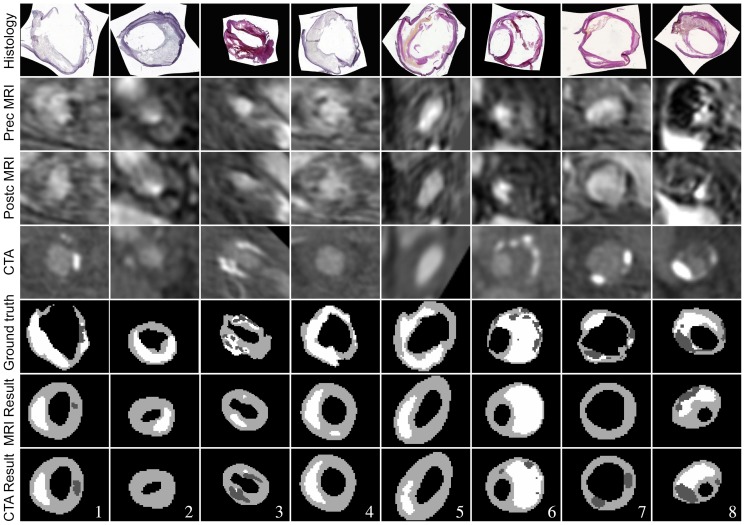
Segmentation results when only MRI, or only CTA, and distance features are used. Results are obtained including outlier rejection. White = LRNC, light gray = fibrous tissue and dark gray = calcification.

Examples of slices segmented using either MRI or CTA and distance features are shown in Figure 6. These show indeed that calcification spots are not accurately detected in MRI (in 1, 3 and 6–8), and that LRNC areas are better segmented when MRI is used (The relative volume is more accurate in 1–3, 5 and 6).

### Relevance of features

To give an indication of the most relevant features, the first five features selected by forward feature selection with LDC are provided in Table 5. It is clear that CTA intensity was the most important feature to segment calcification, and that distances and the combination of pre- and postcontrast images performed best to segment fibrous and lipid-rich necrotic tissue. Also first and second order derivatives showed to be relevant. This does not mean that these are the individually best performing features. For example, to differentiate fibrous tissue and LRNC, the individual features at position 2–6 (2DT1w blurred, lumen distance, PDw blurred, 3DT1w postcontrast GM and 2DT1w precontrast) that gave individually the highest LDC accuracy, were not found in the top 5 with forward feature selection.

**Table 5:** Feature selection.

Calcification-Fibrous	Calcification-LRNC	Fibrous-LRNC
CTA intensity	CTA intensity	Distances multiplied
Distance to lumen	Distances multiplied	TOF Gradient Magnitude
3DT1w pre-contrast Laplacian	3DT1w pre-contrast Laplacian	3DT1w post-contrast Blurred
TOF Gradient Magnitude	Distance to lumen	3DT1w pre-contrast Blurred
PDw blurred	3DT1w post-contrast Blurred	3DT1w post-contrast Laplacian

This table gives the first five features selected by forward selection using LDC accuracy as evaluation criterion, for the separation of each combination of two classes.

## Discussion

We segmented different components of atherosclerotic plaques using both *in vivo* MRI and CTA images, by training a voxelwise classifier on labels obtained from registered histology and 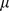
CT and taking into account the presence of misregistrations. Three different approaches (blurring and weighting by Dice overlap, weighting by contour distance, and Gaussian outlier rejection) showed that taking registration errors into account can improve component volume estimations in certain situations. In addition, we showed that combining MRI and CTA images results in better segmentations than when only MRI or only CTA is used.

For LDC no change was observed when sample weights were based on the local contour distances compared to the hard labels, but the bias for fibrous tissue and LRNC became smaller after blurring and weighting by the Dice overlap, and both the bias and absolute error decreased using Gaussian outlier detection. For SVM all three approaches improved the error and correlation for fibrous tissue and LRNC, but decreased classification accuracy for calcification. Weighting by the Dice overlap has a relatively large effect on the class priors (5% (Calcification), 56% (Fibrous) and 38% (LRNC) for hard labels, and 5%, 49% and 46% for weighting with Dice^24^), in contrast to weighting by contour distance and outlier rejection. This may have caused the change in bias of LRNC and fibrous tissue for LDC. Gaussian outlier rejection and LDC are both based on estimation of mean and covariance of Gaussian distributions, which may explain why the combination performs well. The advantage of outlier rejection was especially present in slices with a larger lipid content, which are the slices with more challenging composition that are more difficult to segment. For SVM, samples on the decision boundary determine the final segmentation result, which can be a reason why changing the weights based on local contour distance works better in this situation. LDC has been used previously for plaque component segmentation [20], [36], and performed better than SVM in our experiments. This indicates it is indeed a suitable classifier for this problem.

To compare our results with previous studies Table 6 can be used. Two previous studies automatically segmented plaque components in *in vivo* MRI and compared their results with histology [18], [19]. These show Pearson correlation values (*R^2^*) of 0.83 for calcifications, 0.78 for necrotic tissue, 0.41 for loose matrix and 0.82 for fibrous tissue [18], and (*R*) 0.41 for calcifications, 0.75 for lipid, 0.61 for hemorrhage and 0.67 for fibrous tissue [19], compared to our values (R, see Table 6) of 0.92 (calcification), 0.78 (fibrous) and 0.79 (LRNC) using both MRI and CTA. In [20] plaque component segmentation results were compared with manual annotations of the *in vivo* data, which gave correlation values (R) of 0.88 for lipids, 0.80 for hemorrhage and for fibrous tissue and 0.10 for calcification. Although results are difficult to compare, our results are in a similar range. Hofman et al. [19] obtained less accurate classification results, but this study did not use any spatial information such as distance to the vessel wall. The method by Liu et al. [18] yielded high accuracies and is available within a commercial software package for plaque analysis [54]. This method is also based on voxel classification, and is followed by a level-set segmentation, resulting in more smoothly segmented regions. Spatial regularization is in our case achieved by using Gaussian features and distances and we found this leads to spatially coherent segmentations. The higher accuracies by [18] can be caused by the use of histology-guided manual contours. These are based both on histology and on known MRI intensities, which may bias the segmentations towards intensity (gradients) seen in the MRI. A recent study [24] has shown that the LRNC appears smaller on MRI images than in histology, which is in correspondence with our segmentation results.

**Table 6:** Comparison to previous studies.

Study	Data	Evaluation	Results
Our study	13 subjects (144 slices) MRI and CTA Leave-one-subject-out cross-validation	Histology % of total volume per vessel	Calcium: 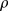 = 0.91, R = 0.92, ICC = 0.92 Fibrous: 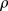 = 0.78, R = 0.78, ICC = 0.76 LRNC: 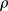 = 0.81, R = 0.79, ICC = 0.76
Liu et al., 2006 [18]	12 subjects (58 slices) MRI 14 subjects (84 slices) for training	Histology-guided manual contours Area (mm^2^) per slice	Calcium: R^2^ = 0.83 Fibrous: R^2^ = 0.82 Loose matrix: R^2^ = 0.41 Necrotic: R^2^ = 0.78
Hofman et al., 2006 [19]	13 subjects (89 slices) MRI 12 subjects for training	Histology % of total volume per vessel	Calcium: R = 0.44 Fibrous: R = 0.69 Lipid: R = 0.74 Hemorrhage: R = 0.63
van't Klooster et al., 2012 [20]	40 subjects (344 slices) MRI 20 subjects for training	Manual annotations Volume (mm^3^) per vessel	Calcium: R = 0.1, ICC = 0.1 Fibrous: R = 0.8, ICC = 0.8 Lipid: R = 0.88, ICC = 0.65 hemorrhage: R = 0.8, ICC = 0.8
Wintermark et al., 2008 [16]	8 subjects (53 slices) CTA	Histology presence/absence 212 quadrants of 53 slices	Calcium: all correct Lipid: 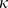 = 0.495 (large areas: 0.796) Large hemorrhage: 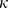 = 0.712 Ulceration: 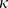 = 0.855
De Weert et al., 2006 [15]	14 subjects (41 slices) CTA	Histology % of area per slice	Calcium: R^2^ = 0.74 Fibrous: R^2^ = 0.76 Lipid: R^2^ = 0.24

Similar to our experiments in which we only used MRI, previous studies that used MRI for segmentation, found low correlations for calcification [19], [20], except for the paper by Liu et al. [18]. Although calcification is difficult to detect in MRI, in Figure 6 dark spots can be seen at calcified locations. Reasons for poor detection in our study are low visibility in other slices, noise, dark-appearing artefacts that do not represent calcifications, and small misregistrations in the training data. The study of Liu et al. [18] obtained their ground truth by histology-guided manual annotation which eliminates the effect of misregistration. MRI sequences that are more specifically aimed at visualizing calcium, could also improve its detection [55].

Two previous studies compared automatic segmentation results in CTA with histology [15], [16], based on a fixed intensity threshold on the CTA to separate LRNC from fibrous tissue and fibrous tissue from calcification. Both these studies accurately segmented calcifications, but obtained lower accuracies for LRNC. Although de Weert et al. [15] found a significant difference between Hounsfield units for lipid (25
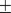
19) and fibrous tissue (88
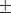
18), the correlation for lipid volume is low (*R^2^* = 0.24, fibrous 0.76 and calcification 0.74). In mildly calcified (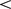
10%) plaques the correlation for LRNC increased (*R^2^* = 0.77), which they relate to the blooming effect of calcification which may overshadow parts of soft plaque. Wintermark et al. [16] found overlapping Hounsfield units for lipid (32.6
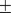
20.0) and connective (fibrous) tissue (46.4
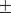
19.9). Concordance between CTA and histology in the detection of lipid tissue was therefore low (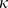 = 0.495), but increased when only large lipid cores were included (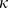 = 0.796). The difficulty they encountered to accurately segment lipid volumes was also observed in our study, which showed large errors when only CTA was used. In our experiments, the reasonable correlation with the ground truth for fibrous and lipid-rich necrotic tissue seems to be mostly based on the distances to the lumen and outer vessel wall, which on itself already yield plausible segmentations. Blooming artefacts in CTA have probably caused the overestimation of calcification when only CTA intensity was used. Blooming in the 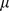
CT did not affect the ground truth due to the relatively high resolution compared to the *in vivo* resolution.

Scanning patients with both MRI and CTA puts a higher burden on both patients and healthcare costs and therefore the combination may not seem relevant in practice. However, in most cases patients that enter the hospital with symptomatic carotid artery disease are already scanned with CTA [56], [57]. MRI has no ionizing radiation, so is relatively safe for patients and can better determine the extent of non-calcified components of vulnerable or complicated plaques [58], [59]. Clinical studies showed that MRI and CTA have different advantages and that combining them may allow for more accurate decision on plaque vulnerability and treatment planning [21], [22]. Recently, clinical studies are emerging that perform both CTA and MRI imaging, showing the clinical possibility and relevance [60]–[62]. Ultrasound is another imaging modality that is feasible for imaging the carotid artery, due to its low cost and wide availability. Therefore ultrasound seems especially useful for screening. It can be used to study plaque vulnerability [63], but accurate quantification of plaque components is not possible due to the limited contrast [64]. For an accurate study of the vessel wall in high-risk patients MRI and CTA are recommended.

When the proposed segmentation framework is to be used for analysis of a new patient several steps need to be taken. These steps with their computation time on a desktop computer (2.26 GHz, 12.0 GB RAM) are: registration of MRI and CTA data (5 registrations, 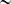
0.5 minute per registration) within a mask (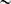
1–2 minutes for annotation on the CTA and T1w postcontrast MRI each), manual lumen and outer wall segmentation (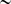
10 minutes, but this can be automated [65], [66]), inhomogeneity correction (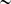
10–15 seconds per MR image), feature computation (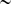
4–5 seconds), applying the classifier and obtaining segmentations (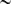
1 second).

Our current results show a good Spearman rank correlation for the amount of LRNC, which is an indication of plaque vulnerability [67]. Whether this can reliably be used to select high-risk patients for treatment can, however, not be determined in this study. This has to be determined in a large group of patients that are followed for clinical events, followed by a clinical trial where the advantage of using plaque composition for treatment selection is evaluated. In our results the stroke patients had a LRNC of 44% (range 10–71), and the patients who had a TIA had a LRNC of 27% (22–46). The amount of calcification was 3% (0–6) after stroke, and 7% (1–11) after TIA. The automated results yielded similar results: a LRNC of 46% (1–64) and 23% (5–29), and calcifications of 3% (0–6) and 5% (1–13) for stroke and TIA patients respectively. This corresponds to the idea that a LRNC characterizes more vulnerable plaques and calcifications characterize more stable plaques [6], [7]. The asymptomatic patient in this study had a LRNC of 20% and 1% of calcification.

This work has several limitations. There was a considerable time interval between the MRI and CTA scan (38
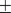
26 days), but we do not expect noticeable changes in plaque composition and volume to occur in this period. Previous studies did not find changes in wall and component volumes or presence in a mean of 12–15 days [68], [69] or a year [70].

For this study no histology sections were stained to specifically detect intraplaque hemorrhage (IPH), and hemorrhage was therefore not included as a separate component. In addition, the used T1w MRI sequences are not the most suitable for imaging of IPH [71]. Still, based on hyperintensity in the precontrast 2D- and 3D-T1w MR images, we found a suggestion of IPH presence in 5 vessels (4 with stroke, 1 with TIA). For all these vessels this area was included in the LRNC segmentation. The error for LRNC was on average not larger in those 5 vessels than in the other 8 for the experiments including MRI and/or CTA and distance features. When only CTA intensity was considered no LRNC was segmented, both when all patients were included and when only the patients without possible IPH presence were included in training and evaluation. This suggests that IPH presence is not causing large errors, nor explains the poor performance of CTA in this study. Using the presented approach, hemorrhage can be easily added separately to the framework when a ground truth is available, and its inclusion would be highly valuable in future studies [7]. We also did not differentiate between fibrous and loose connective tissue as was done in [18], as these are difficult to distinguish in histology and both are stable plaque components. Before histology sectioning the specimens were decalcified, and during sectioning areas of lipid tissue may be disrupted, which could cause mixing up the two tissues. However, in the empty regions in the histology sections we could distinguish calcium from lipid by using the 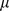
CT as a reference.

In this work we evaluated our results based on relative plaque component volumes. Ideally, the classification accuracy would be evaluated in a voxelwise manner. We could not do this because no accurate voxelwise correspondence could be established between *in vivo* data and histology, even with substantial manual interaction. As a surrogate measure, we chose relative plaque component volumes as they provide a clinically relevant biomarker for prediction of plaque vulnerability [67], [72]–[74]. Additionally, there were only 13 subjects included in this study. However, due to the challenges involved in plaque excision, sectioning and staining, it was not possible to add more vessels.

Lastly, in this work we use manual annotations of the vessel wall in *in vivo* images. Several automated vessel wall segmentation methods are available [54], [65], [66], [75]. The combination of automated wall segmentation with component segmentation would yield a highly automated plaque analysis tool. As long as an automatic wall segmentation is accurate, and possibly manually adjusted in case of errors, we expect using an automatic segmentation method has no influence on the results described in this paper.

## Conclusion

The volume of atherosclerotic plaque components can be well estimated using a classifier trained on histology. Different approaches to account for mismatch between the imaging data and the ground truth from histology can slightly improve segmentation. While MRI can better differentiate between fibrous and lipid-rich necrotic tissue, and CTA can better segment calcification, the combination leads to good results for all three components. This can facilitate the use of quantitative plaque composition in large clinical studies and possibly future patient risk assessment.

## Supporting Information

Movie S1 This supporting information movie displays segmentation results for all 13 vessels, in addition to the segmentation of one vessel that is provided in Figure 5.
(ZIP)
